# Pulsed supplies of small fish facilitate time-limited intraguild predation in salmon-stocked streams

**DOI:** 10.1098/rsos.220127

**Published:** 2022-09-21

**Authors:** Koh Hasegawa, Sho Fukui

**Affiliations:** Salmon Research Department, Fisheries Resources Institute, Japan Fisheries Research and Education Agency, Nakanoshima, Toyohira, Sapporo, Hokkaido 062-0922, Japan

**Keywords:** density dependence, diet niche, hatchery program, interspecific competition, piscivorous, size dependence

## Abstract

Pulsed supplies of prey generally increase predator food intake. However, it is unclear whether this holds true when predators and pulsed prey are in the same guild (i.e. intraguild (IG) predators and prey). IG prey may increase IG-predator food intake through predation, but they may decrease food intake through competition. To test these hypotheses, we compared the food intake of white-spotted charr (*Salvelinus leucomaenis*) (IG predator) in streams that were stocked or unstocked with masu salmon (*Oncorhynchus masou*) fry (IG prey) in streams in Hokkaido, Japan. One day after stocking, mean stomach content weight of charr was six times higher than in unstocked streams due to fry consumption. In particular, large charr showed intense piscivory. However, predation on fry was rare after about three weeks. Some factors that could explain this time-limited IG predation include the growth and decreasing abundance of fry over time and the acquisition of predator-avoidance behaviour. In days other than the first-day post-stocking, food intake by charr did not differ between stocked and unstocked streams. No effects of interspecific competition on charr food intake were observed.

## 1. Introduction

Pulsed supplies of individuals or organisms can dramatically affect recipient ecosystems by facilitating predation, increasing competition and altering diets and food intake [1]. One common example of pulsed supply is masting, in which individuals of one plant species synchronously produce large amounts of seeds in the same season, and consumers (e.g. rodents) exclusively forage on the seeds in competition with other consumers [2,3]. Outbreaks of the desert locust (*Schistocerca gregaria*) during great migrations are another typical example: the locust swarms decrease food intake of indigenous herbivores through resource competition, while increasing the food intake of predators [4].

Intraguild (IG) predation occurs when predators (IG predators) prey upon potential competitor species (IG prey) [5]. The occurrence of IG predation is affected by the relative density of IG prey and of shared food resources (i.e. the total food availability for IG predators) [6], and sometimes by body size differences between IG predators and IG prey [7]. Pulsed supplies of IG prey or of shared food resources can modify IG predation relationships by changing these relative densities [6]. For example, pulsed supplies of shared food resources (an outbreak of rodents) decreased predation pressure by IG predators (the dingo [*Canis dingo*]) on IG prey (the red fox [*Vulpes vulpes*] and feral cat [*Felis catus*]) in the Australian desert [8]. The effects of a pulsed supply of IG prey, however, are likely to be more complex. Since IG prey function as both competitors and prey for IG predators, a pulsed supply of IG prey may decrease food intake and growth of IG predators through resource competition, while at the same time increasing food intake by boosting the supply of prey resources.

One example of an artificially pulsed supply of organisms is the stocking of hatchery-reared fish into natural environments. The stocking of hatchery-reared salmonid fry, in particular, is frequently conducted in rivers inhabited by other wild salmonids. Because both hatchery-reared and wild salmonids predominantly prey upon aquatic and terrestrial invertebrates, they are often in competition for food resources [9]. In salmonids, both interspecific competition and competition between hatchery-reared and wild fish are density dependent [10,11]. Interference competition, in which dominant (i.e. larger) individuals occupy profitable foraging territories, is the principle competitive mode, and exploitative competition, in which individuals scramble for food resources, is thought to occur simultaneously [12]. If multiple salmonid species coexist in the same stream, their dietary niches may be affected by interspecific competition. Diet niche partitioning is sometimes regarded as the outcome of interspecific competition (e.g. one species mainly preys upon drifting terrestrial invertebrates, while another preys upon benthic invertebrates [13,14]). Although high diet niche overlap is often indicative of intense interspecific competition [15], it may also occur in systems where one type of food item is highly abundant, and fish prey upon them exclusively. In such systems, density-dependent competition may be lacking.

Salmonids prey on fish to the extent that their gape size and prey size allow [16–18]. Habitat types can also influence the frequency of piscivory. Of the three types of habitats inhabited by salmonids (riverine, lacustrine and marine habitats), the occurrence of piscivory is likely to be the lowest in riverine habitats due to the high availability of other food items such as drifting aquatic and terrestrial invertebrates [19,20]. By contrast, riverine salmonids will still prey exclusively upon stocked salmon fry just after stocking (i.e. when there is an extremely high availability of stocked fry) [21]. However, predation on stocked fry may decline over time as fry growth increasingly provides a size refuge from predation.

Therefore, streams stocked with hatchery-reared salmonid fry are ideal experimental systems in which to test hypotheses regarding body size-dependent IG predation and the pulsed supply of organisms. In this study, we conducted field surveys to test the following hypotheses: (1) IG prey supplied pulse-wise will compete for food resources with IG predators; (2) large IG predators will increase their food intake through IG predation and (3) the frequency of IG predation will decrease with increased fry (i.e. IG prey) body size.

## 2. Materials and methods

### 2.1. Study systems

**Figure 1.**
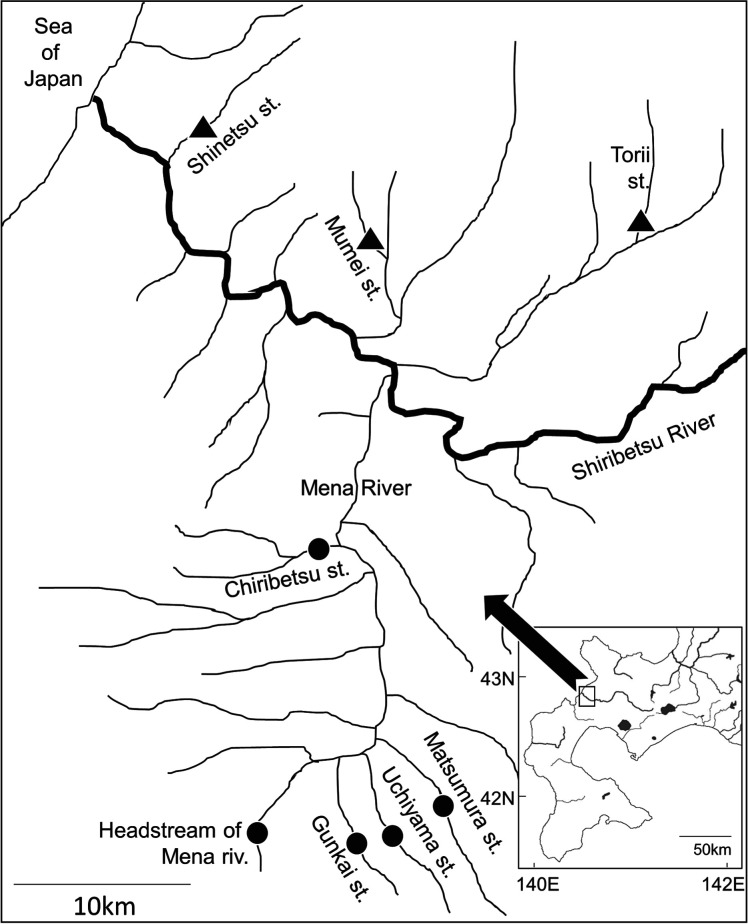
Locations of each study site in the Shiribetsu river system. Circles and triangles indicate study sites for stocked and unstocked streams, respectively.

A field survey was conducted in 2019 in wadeable tributaries of the Shiribetsu River in Hokkaido, northern Japan (figure 1). Stocking of hatchery-reared masu salmon (*Oncorhynchus masou masou*) has been conducted in the Mena River, a large tributary of the Shiribetsu River, since 1915 [22]. In our survey year, 900 000 masu salmon fry were stocked in this tributary in total. Four stocked survey sites were established in the Mena River system to detect the effects of stocked fry (figure 1, table 1). All fry stocking in these streams was conducted on 29 May. Masu salmon stocked in the preceding year were rarely observed at these sites. In addition to the four stocked sites, three control sites (no stocked fry) were established in other tributaries of the Shiribetsu River (triangle symbols in figure 1). In this study, the stocked fry were treated as IG prey. Potential IG predators across the seven study sites were white-spotted charr (*Salvelinus leucomaenis*), masu salmon and sculpin (*Cottus nozawae*). Of these, white-spotted charr were common to all study sites and were therefore deemed the target IG predators. A previous study demonstrated that white-spotted charr frequently preyed upon masu salmon if the fork length of the masu salmon was less than 40% that of the white-spotted charr [23]. Water temperatures in the study sites were typical for streams inhabited by salmonids (electronic supplementary material, table S1).

**Table 1.** Length and mean width measured at 10 points of each study site. Numbers of fry stocked on 29 May and fishes captured by one-pass electrofishing for each study site and study period are shown (top: stocked masu salmon fry; bottom: white-spotted charr). In late May, the actual numbers of fry were uncountable, but more than 1000 fry were certainly captured.

				number of fishes captured				
study sites	no. of fry stocked (×1000)	length (m)	mean width (m)	mid-May	late May	mid-Jun	late Jul	ear Sep
<stocked>				before	after stocking			
				stocking				
headstream of	130	128	2.0	−	>1000	265	124	36
Mena riv.				10	6	11	8	8
Gunkai	30	110	2.0	−	>1000	339	161	81
stream				2	4	2	4	5
Uchiyama	30	65	2.0	−	>1000	117	96	67
stream				3	15	8	15	12
Matsumura	30	62	2.9	−	>1000	141	111	76
stream				15	17	26	22	16
<unstocked>								
Shinetsu		179	5.2	−	−	−	−	−
stream				5	13	14	14	12
Mumei		73	4.0	−	−	−	−	−
stream				9	8	10	11	9
Torii		94	2.3	−	−	−	−	−
stream				10	17	25	38	20

Footnotes: Dates for each study period were as follows: mid-May: 13–15 May; late May: 29–31 May; mid-Jun: 18–20 June; late Jul: 22–24 July; ear Sep: 3–4 September.

### 2.2. Sampling procedures

Sampling was conducted once prior to stocking, and four times after stocking (table 1). In stocked streams, sampling was conducted on the day after stocking in late May. To capture fish, a sampler walked upward from the downstream margin of each study site while using a backpack electrofisher (Smith-Root Inc., Vancouver, WA, USA). The numbers of each fish species captured were used as indices of fish density. Although stocked fry in late May were too numerous to count, we confirmed that there were a few thousand fry at each site, and that they were obviously more abundant than during other study periods.

Captured fish were anaesthetized by using ethyl 3-aminobenzoate methanesulfonic acid, and their fork lengths were measured to the nearest 1 mm. There were no significant differences in the mean fork lengths of white-spotted charr between study periods or between stocked and unstocked study sites (electronic supplementary material, table S2).

After measuring the fish for fork length, we sampled the stomach contents of white-spotted charr (up to 17 individuals per study site) by gastric lavage, and of stocked fry (15 individuals per study site) by laparotomy in the laboratory. Stomach contents and fry were immediately fixed in 70% ethanol. In the laboratory, the wet weight of each diet item in the stomach contents was determined to the nearest 0.001 g. Invertebrates were separated into terrestrial and aquatic invertebrates, and aquatic invertebrates were identified to the family or genus.

Except for the stocked fry from the laparotomy, captured fish were released to each study site. White-spotted charr were tagged with a banok tag at the time of release, which allowed us to evaluate growth rates by comparing fork lengths between initial and subsequent captures. In addition to the four stocked streams, charr tagging was conducted in the Chiribetsu stream in which also masu salmon fry were stocked to supplement the growth data (figure 1).

### 2.3. Data analysis

Diet niche overlaps between white-spotted charr and stocked masu salmon fry were quantified by using a proportional similarity index (PS) [24] for each study site where fry were stocked:

2.1 $$\mathrm{PS}=1-0.5\overset{m}{\underset{i=1}{\sum }}|\mathrm{WC}i-\mathrm{MS}i|,$$

where WC*i* and MS*i* represent the wet weight proportions of prey category *i* (of *m* categories) for white-spotted charr and stocked masu salmon fry, respectively. The category ‘other aquatic invertebrates’ was excluded in this calculation. The index ranged from 0 (no overlap) to 1 (complete overlap). Temporal changes of PS were tested by using Friedman's test.

A linear mixed model with piscivory/non-piscivory as a fixed effect and study site as a random effect was used to compare the fork lengths of white-spotted charr that preyed upon stocked masu salmon fry in late May and those that did not.

To compare stomach content weights of white-spotted charr between stocked and unstocked study sites, a general linear model was constructed as follows:

2.2 $$\begin{array}{r} \mathrm{Stomach}\;\mathrm{content}\;\mathrm{weight}∼\mathrm{study}\;\mathrm{period}+\mathrm{stocked}/\mathrm{unstocked}\;(\;\mathrm{study}\;\mathrm{site})\;\\ \;+\mathrm{study}\;\mathrm{period}\;\times \mathrm{stocked}/\mathrm{unstocked}\;(\;\mathrm{study}\;\mathrm{site})\;, \end{array}$$

where stomach content weights were specified for each experimental fish. Study periods were treated as a categorical valuable. Study site was nested within stocked/unstocked streams.

Specific growth rates (SGRs) of recaptured charr were calculated as follows:

2.3 $$\mathrm{SGR}=\frac{100(\;\mathrm{lnF}L_{1}\text{–}\mathrm{lnF}L_{0})}{t},$$

where FL_0_ and FL_1_ are fork length on the days of tagging and recapture, respectively, and *t* is the days elapsed between these procedures.

SGRs between late May and mid-June were calculated for 10 charr recaptured at study sites at which masu salmon fry were stocked and for 13 charr recaptured at sites where they were not. SGRs were compared between stocked and unstocked streams with the following general linear model:

2.4 $$\begin{array}{r} \mathrm{SGR}∼\mathrm{stocked}/\mathrm{unstocked}\;(\;\mathrm{study}\;\mathrm{site})+\mathrm{fork}\;\mathrm{length}\\ \;+\mathrm{fork}\;\mathrm{length}\;\times \mathrm{stocked}/\mathrm{unstocked}\;(\;\mathrm{study}\;\mathrm{site})\;, \end{array}$$

where fork length in late May was treated as a covariate because large individuals could achieve high growth through competitive dominance and by preying upon stocked masu salmon fry [12,16–18]. Study site was nested within stocked/unstocked streams.

Fork lengths of white-spotted charr were log transformed for the analyses. All statistical tests were performed in SPSS version 24 (IBM Corp., Armonk, NY, USA) with *α* = 0.05.

## 3. Results

### 3.1. Number of fish captured and diet niche overlap

**Figure 2.**
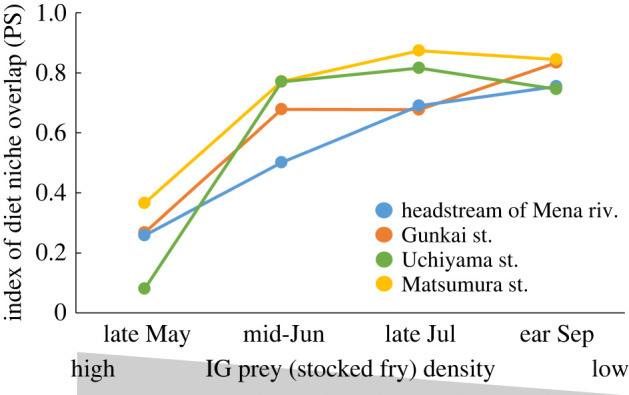
Time series of diet niche overlap between white-spotted charr and stocked masu salmon fry as indicated by the proportional similarity index [24]. The grey shaded area below the horizontal axis is a conceptual representation of the decline in stocked masu salmon density over time.

The number of stocked masu salmon fry caught by single-pass electrofishing gradually declined over time, and the number of white-spotted charr did not show a noticeable trend (table 1). Additionally, stocked masu salmon fry vastly outnumbered white-spotted charr in stocked streams throughout the survey (table 1). Because of piscivory by white-spotted charr, the degree of diet niche overlap in late May was smaller than during other study periods (Friedman's test: d.f. = 3; *χ*^2^ = 9.96, *p* = 0.019) (figure 2).

### 3.2. Piscivory of white-spotted charr

**Figure 3.**
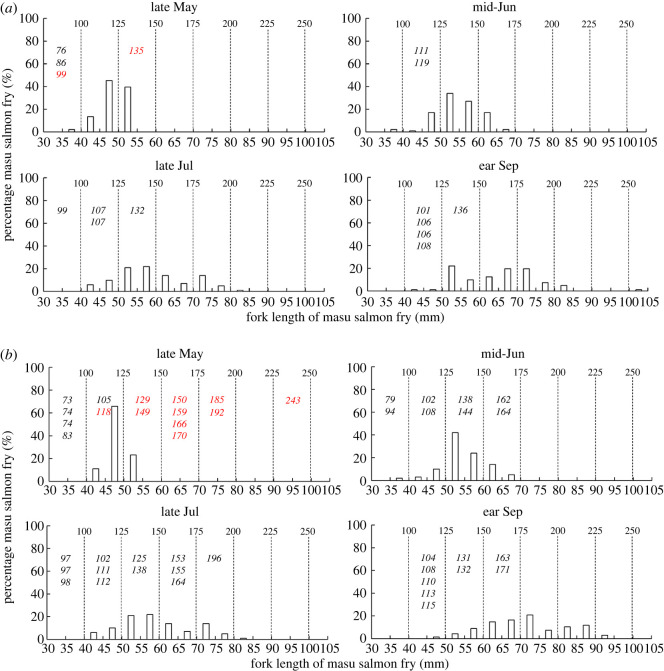

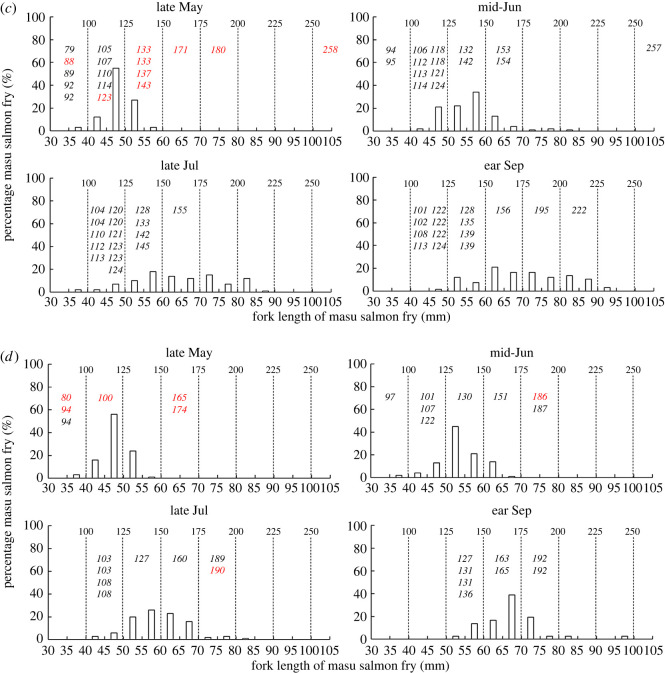
Body size (fork length: mm) histograms of stocked masu salmon fry and fork length (mm) of each experimental white-spotted charr in each stocked stream (*a*: Gunkai; *b*: Uchiyama; *c*: Matsumura; *d*: headstream of the Mena River) in each study period. Values shown in italics show fork lengths of white-spotted charr that did (red) and did not (black) prey on masu salmon fry. Bold values above the dashed lines indicate the theoretical minimum white-spotted charr fork length needed to prey upon masu salmon fry of the fork length [23] shown below the dashed line.

In stocked streams, all fish identified in the stomach contents of white-spotted charr were stocked masu salmon fry. Also, 26 out of 42 white-spotted charr examined preyed upon at least one masu salmon fry in late May (figure 3). White-spotted charr which preyed upon masu salmon fry were significantly larger than those that did not prey upon the fry (linear mixed model: *F*_(1, 38)_ = 38.56, *p* < 0.001) (figure 3). After late May, the body size (fork length) of stocked masu salmon fry increased, and masu salmon fry with small body size, which some small white-spotted charr were able to prey upon gradually decreased (figure 3). In addition, even white-spotted charr that were large enough to prey upon masu salmon fry seldom engaged in piscivory after mid-June (only 2 of 110 white-spotted charr preyed upon masu salmon fry) (figure 3). No sampled white-spotted charr preyed upon fish in unstocked streams.

### 3.3. Temporal changes of stomach content weight of white-spotted charr

**Figure 4.**
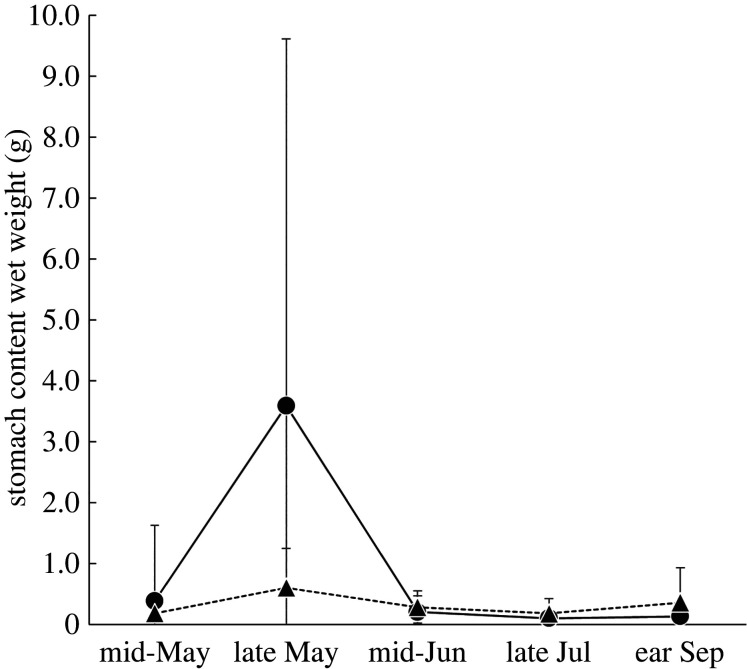
The comparison of mean stomach content wet weight of white-spotted charr between stocked and unstocked streams throughout the survey. Error bars indicate s.d. Sample sizes are shown in the electronic supplementary material, table S2.

The significant interaction term suggests that differences in stomach content weight between stocked and unstocked streams varied across study periods (table 2). Although stomach content weights for stocked streams were nearly six times larger than for unstocked streams in late May, the difference between the two stream types was not clear during other study periods (figure 4).

**Table 2.** The result of general linear model testing the effects of study periods and stocked/unstocked stream, and their interaction term on the stomach content weight of white-spotted charr. Study sites are nested within stocked/unstocked.

	d.f._numerator_	d.f._denominator_	*F*	*p*
study periods	4	547	0.522	0.719
stocked/unstocked (study site)	6	547	11.53	<0.001
study periods × stocked/unstocked (study site)	24	547	1.855	0.008

### 3.4. Diet items of white-spotted charr and masu salmon

**Figure 5.**
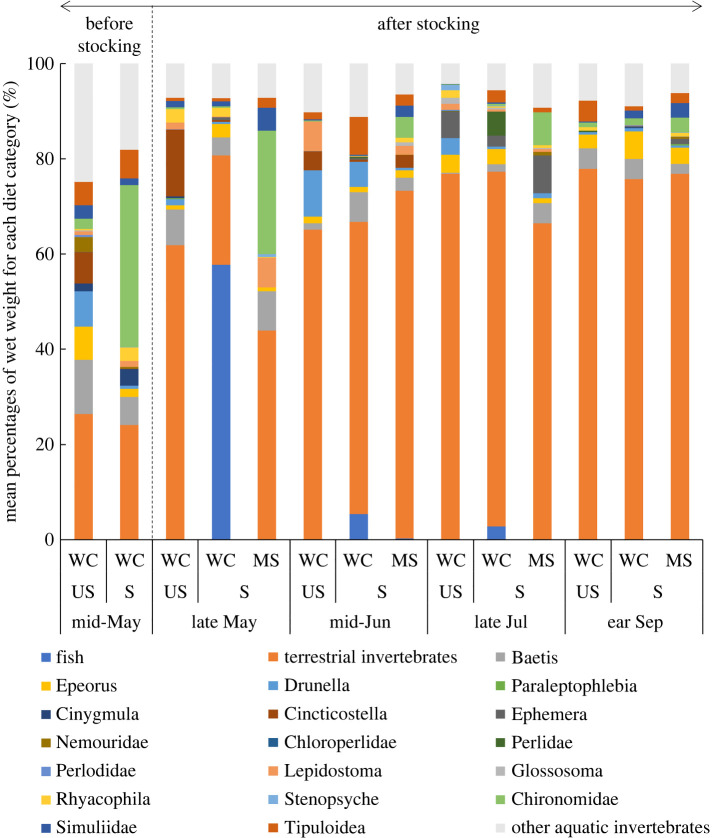
Mean percentages of wet weight for each diet category (see legend for category names) in the stomach contents of white-spotted charr (WC) and stocked masu salmon fry (MS) in stocked (S) and unstocked (US) streams throughout the survey. The wet weights of each diet category sampled from each experimental fish were pooled to calculate the percentages for stocked and unstocked streams. Sample sizes of white-spotted charr are shown in the electronic supplementary material, table S2. For stocked masu salmon fry, there were 60 sampled fish (15 fish × 4 study sites) for each study period.

Diet items were classified into 21 categories (including ‘other aquatic invertebrates’, figure 5). In mid-May, the wet weight percentage of aquatic invertebrates in the diets of white-spotted charr was larger than that of terrestrial invertebrates in both stocked and unstocked streams. From late May to early September (i.e. after fry stocking), the percentage of terrestrial invertebrates exceeded 60% in all streams, with the exception of stocked streams in late May. In stocked streams in late May (i.e. immediately after fry stocking), masu salmon fry accounted on average for nearly 60% of the wet weight of individual white-spotted charr stomach contents. Among white-spotted charr with fish in their stomach contents (i.e. the larger charr; figure 3), masu salmon fry accounted for 84.4% on average (range 26.1%–100%) of the wet weight of stomach contents. Meanwhile, non-piscivorous white-spotted charr (i.e. the smaller charr; figure 3) mainly preyed upon terrestrial invertebrates, and terrestrial invertebrates accounted for 67.4% on average (range 16.7%–100%) of the wet weight of the stomach contents. Like white-spotted charr, stocked masu salmon fry mainly consumed terrestrial invertebrates (figure 5). Of the aquatic invertebrates, three categories of mayfly larvae (genus *Baetis*, *Epeorus* and *Drunella*) were dominant in the benthic communities of both stocked and unstocked streams throughout the survey (electronic supplementary material, figure S1), and they were also frequently identified in the stomach contents of masu salmon fry and white-spotted charr in both stocked and unstocked streams (figure 5).

### 3.5. Specific growth rates of white-spotted charr

**Figure 6.**
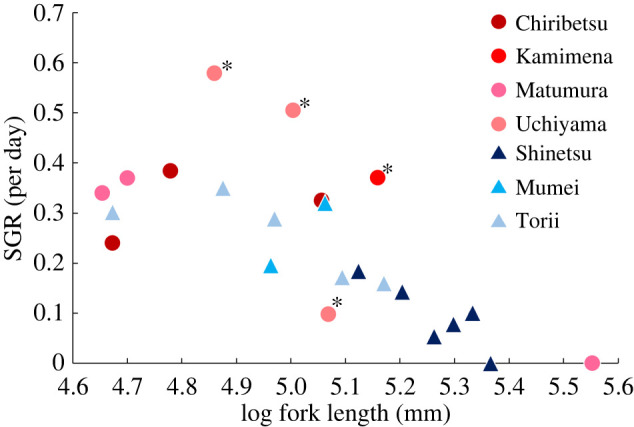
Relationships between fork length (in late May) and SGR (from late May to mid-June) of white-spotted charr. Each point indicates a single individual. Red circles indicate fish in stocked streams, and blue triangles indicate those in unstocked streams. Asterisks indicate individuals that preyed on masu salmon fry in late May.

The interaction term was marginally significant, suggesting that relationships between fork length and SGR differ between stocked and unstocked streams (table 3). Additionally, the SGR tended to be larger in stocked streams than that in unstocked streams (figure 6).

**Table 3.** The result of general linear model testing the effects of log transformed fork length at late May (covariate) and stocked/unstocked stream, and their interaction term on the SGRs of white-spotted charr. Study sites are nested within stocked/unstocked.

	d.f._numerator_	d.f._denominator_	*F*	*p*
stocked/unstocked (study site)	5	10	2.918	0.070
fork length	1	10	2.034	0.184
stocked/unstocked (study site) × fork length	5	10	2.870	0.073

## 4. Discussion

Stocked masu salmon fry are a potential competitor of white-spotted charr regardless of their body size difference, as is evidenced by their similar dietary niches, which included terrestrial invertebrates and mayfly larvae. However, the consumption of stocked masu salmon fry contributed to an increase in the stomach contents of white-spotted charr, especially the larger individuals. This suggests that IG predation occurred depending on the relative body size difference between IG predators and IG prey [23], and importantly the pulsed supply of IG prey can contribute to an increase in the food intake of IG predators, despite the simultaneous interspecific competition for food resources. However, the occurrence of IG predation in our study was time-limited.

Even though salmonids are known to exhibit intense density-dependent competition which decreases food intake and/or growth of individuals with increasing density [10], the peak of stocked masu salmon fry density in late May coincided with the peak of white-spotted charr food intake in our study. Interspecific competition often drives niche partitioning in many salmonids [13,14], and diet niche partitioning between white-spotted charr and stocked masu salmon fry was the most noticeable in late May. This diet niche partitioning was clearly caused by predation of the larger white-spotted charr on stocked masu salmon fry. By contrast, the smaller white-spotted charr which did not show piscivory and stocked masu salmon fry both preyed upon mainly terrestrial invertebrates. This suggests that small white-spotted charr and stocked masu salmon fry were competitors without IG predation. Given these factors, the co-occurrence of body size-dependent IG predation with interspecific competition could cause researchers to misinterpret patterns of food intake of white-spotted charr as being caused entirely by density-dependent competition.

The time-limited occurrence of predation on stocked salmonid fry by piscivorous salmonid species has been reported in a previous study [25]. During our study, stocked masu salmon fry became less numerous and began to outgrow the gape limitation of small white-spotted charr. These two factors could be partly responsible for the time-limited occurrence of predation. A similar pattern has been observed in Alaskan lakes from June to July, where predation on sockeye salmon (*Oncorhynchus nerka*) fry by coho salmon (*Oncorhynchus kisutch*) decreased with sockeye salmon growth [26]. Nonetheless, there were still large white-spotted charr able to prey upon masu salmon fry after mid-June. Thus, we must consider other causes of the time-limited occurrence of predation. For example, stocked fry could have gradually acclimated to stream environments and learned predator-avoidance behaviour [27]. Additionally, the riverine white-spotted charr that were being examined in our study were likely generally of the insectivorous rather than piscivorous ecotype [28,29]. Thus, their foraging behaviour may not have been effective against masu salmon fry except during the short initial period when stocked fry had not acclimated to the stream environment and were drifting downstream in search of suitable microhabitats. Thus, late May (i.e. several days after stocking) might have been the only time when the white-spotted charr could prey upon stocked masu salmon fry. Predation on masu salmon fry may have partly contributed to the growth of white-spotted charr, although the small sample size of the growth data limits the confidence of this finding.

After mid-June, the food intake of white-spotted charr in stocked streams was similar to that in unstocked streams. Predation was rare during this period, and stocked masu salmon fry were more likely to interact with white-spotted charr as competitors than as prey. However, the density of stocked masu salmon fry and/or the difference in food availability for white-spotted charr in stocked versus unstocked streams may not have been enough to make an impact. In addition, because the body sizes of white-spotted charr were larger than those of stocked masu salmon fry throughout the survey period, interference competition from masu salmon might not have had a negative impact on white-spotted charr [12]. Similarly, because the foraging habitats of salmonids in streams differ by body size (i.e. larger fish use deeper and faster current environments) [30], the two species sampled in this study might not have been in competition for foraging habitat. We were unable to assess the size of ingested prey items in this study due to technical considerations. However, any prey size difference between the two species could have also contributed to a reduction in competition.

Our study demonstrates that pulsed supplies of IG prey can increase food intake among IG predators (through prey consumption), but whether they also decreased food intake of IG predators through interspecific competition is unclear. The seeming absence of strong interspecific competition could have been caused by the body size dependence of IG predation, meaning that the larger white-spotted charr were dominant in any interspecific competition. Any effects of pulsed prey supply on predators in our study were time-limited. In general, prey supplied pulse-wise are not regular food items for predators [3,31], and predators may be unable to accommodate these prey due to mismatches in, for example, foraging behaviour. Finally, our study highlights the importance of ‘ecosystem-based’ fisheries management strategies in which the effects of predator–prey interactions and intra- and interspecific competition on ecosystem structuring are taken into account [32]. Our results provide further clarity on how the supply of salmon fry can alter ecosystem structures in stocked streams.

## Data Availability

Datasets are available on Dryad (https://doi.org/10.5061/dryad.pnvx0k6pk) [33]. The data are provided in the electronic supplementary material [34].
